# Effect of Transfer Film on Tribological Properties of Anti-Friction PEI- and PI-Based Composites at Elevated Temperatures †

**DOI:** 10.3390/polym14061215

**Published:** 2022-03-17

**Authors:** Sergey V. Panin, Jiangkun Luo, Dmitry G. Buslovich, Vladislav O. Alexenko, Filippo Berto, Lyudmila A. Kornienko

**Affiliations:** 1 Laboratory of Mechanics of Polymer Composite Materials, Institute of Strength Physics and Materials Science of Siberian Branch of Russian Academy of Sciences, 634055 Tomsk, Russia; buslovich@ispms.ru (D.G.B.); vl.aleksenko@mail.ru (V.O.A.); rosmc@ispms.ru (L.A.K.); 2 Department of Materials Science, Engineering School of Advanced Manufacturing Technologies, National Research Tomsk Polytechnic University, 634050 Tomsk, Russia; jiangkun169@gmail.com; 3 Department of Mechanical and Industrial Engineering, Faculty of Engineering, NTNU: Norwegian University of Science and Technology, 7491 Trondheim, Norway; filippo.berto@ntnu.no

**Keywords:** polyimide, polyetherimide, transfer film, carbon fibers, polytetrafluoroethylene, molybdenum disulfide, elastic modulus, coefficient of friction, wear rate

## Abstract

The structure, mechanical and tribological properties of the PEI- and PI-based composites reinforced with Chopped Carbon Fibers (CCF) and loaded with commercially available micron-sized solid lubricant fillers of various nature (polymeric-PTFE, and crystalline-Gr and MoS_2_) were studied in the temperature range of 23–180 (240) °C. It was shown that tribological properties of these ternary composites were determined by the regularities of the transfer film (TF) adherence on their wear track surfaces. The patterns of TFs formation depended on the chemical structure of the polymer matrix (stiffness/flexibility) as well as the tribological test temperatures. Loading with PTFE solid lubricant particles, along with the strengthening effect of CCF, facilitated the formation and fixation of the TF on the sliding surfaces of the more compliant PEI-based composite at room temperature. In this case, a very low coefficient of friction (CoF) value of about 0.05 was observed. For the more rigid identically filled PI-based composite, the CoF value was twice as high under the same conditions. At elevated temperatures, rising both CoF levels and oscillation of their values made it difficult to retain the non-polar PTFE transfer film on the sliding surfaces of the PI-based composite. As a result, friction of the ceramic counterpart proceeded over the composite surface without any protecting TF at *T* ≥ 180 °C. For the sample with the more flexible PEI matrix, the PTFE-containing TF was retained on its sliding surface, providing a low WR level even under CoF rising and oscillating conditions. A similar analysis was carried out for the less efficient crystalline solid lubricant filler MoS_2_.

## 1. Introduction

In recent times, due to improved research and knowledge, polymer-based materials are the first choice for materials for industrial applications [1,2,3]. Polyimides (PI) are a class of high-performance polymers (HPP) with a balanced combination of key physical, mechanical and functional properties: improved tensile strength and elastic modulus, great resistance to high temperatures, fire, aggressive environments, radiation and ultraviolet exposure [4,5,6,7,8]. In addition, they can be operated in wide temperature ranges, so PI are widely applied in the aerospace industry, electronics, electrical and transport engineering, etc. Showing exceptionally advanced functional characteristics (close to the maximum achievable for polymeric materials), most PI possess a significant drawback. They are difficult to use as a feedstock for bulk products due to strong intermolecular interactions and high rigidity of polymer chains. Designing both neat PI and PI-based composites, which are processable by conventional high-performance industrial technologies as well as maintaining the basic functional properties, is an urgent task nowadays.

The invention of thermoplastic polyetherimides (PEI) obtained by adding ‘hinged’ oxygen atoms to repeating links consisting of aromatic and heterocyclic fragments was a breakthrough in chemical engineering [9,10]. In this case, the basic PI properties (excellent physical and mechanical characteristics, as well as great onset decomposition temperatures) are retained, and their chain flexibility was enhanced [11]. An increase in the flexibility of polymer macromolecules and/or a decrease in intermolecular interaction open up opportunity for the use of PEI for the fabrication of reinforced composites [12,13,14,15,16].

The improved mechanical properties in wide temperature ranges attract the designers of polymer composites, including those for anti-friction applications. However, neat PI is almost never used for the manufacture of parts for tribological units despite its all-inherent advantages. The reasons are their high strengths and the coefficients of friction (CoF), resulting in significant wear rates (WR) under dry sliding friction conditions [17,18,19]. This issue is solved by loading PI with a number of solid-lubricant fillers, enabling reduction in both CoF and WR parameters. The most common (inexpensive) solid lubricants for filling polymer matrices include polytetrafluoroethylene (PTFE) [20,21], graphite (Gr) [22], molybdenum disulfide (MoS_2_) [23,24], and a number of others. Note that an increase in tribological properties was achieved by loading with solid lubricant fillers of the micron size range.

Several studies of the tribological properties of the PI-based composites in wide temperature ranges were carried out [25,26,27,28,29,30]. In doing so, nanoparticles were widely used as a filler, since their incorporation is generally employed to improve the mechanical performances of polymeric films [31]. In [25], PI-composite loaded with PTFE and multi-walled carbon nanotubes were investigated in the temperature range of 20–240 °C. The authors of [32,33] reported similar data, obtained at *T* = 150 °C, on multicomponent PEI-based composites loaded with short carbon fibers (SCF), Gr, both TiO_2_ and ZnS submicroparticles, as well as CaSiO_3_ microsized inclusions. In the temperature range of 70–150 °C, WR was determined by the contact pressure levels during tribological tests according to the ‘ball-on-disk’ scheme. At the same time, the tribological properties of the PEI-based composites were close to those of the PEEK-based ones loaded with the same fillers. In the latter cases, the glass transition temperatures were much higher (by 10–20%). However, PEI is more preferred for the design of composites for friction units via processing by conventional methods (extrusion, injection molding, etc.) due to its easier processability.

In [34], the authors reported both mechanical and tribological characteristics (at *T* = 23 °C) of ternary commercial PI based composites (PI 1600, Solver) loaded with the commercially available solid lubricant fillers (PTFE, Gr, MoS_2_) and reinforced with chopped carbon fibers (CCF) 2000 µm long. The ‘ball-on-disk’ and ‘block-on-ring’ schemes were used at various (*P·V*) load-sliding speed modes. It was shown that both CoF and WR parameters were controlled by the formation of a transfer film (TF). However, patterns of the formation of such films at elevated temperatures remain only slightly studied yet.

As noted above, both PI and PEI are a class of polymers unified by a common term. Nowadays, there are a large number of commercially available PI and PEI, as well as their composites. Nevertheless, they possess rather different properties despite similar chemical structures, which can be varied substantially, affecting tribological characteristics that also depend on the operating temperatures. Their fundamental difference is that PEI macromolecules are more flexible. So, it is necessary to study samples fabricated by the same manufacturer under comparable conditions in order to clearly reveal the effect of variations in chemical structures, primarily due to the presence of ether rings.

To obtain comparable results of tribological tests for the composites based on two different matrix types, polymer powders from the same manufacturer (Solver, China), as well as the identical commercially available solid lubricant fillers, were utilized. In addition, the tests were carried out under equal conditions. In this work, a comparison of the TF formation patterns was carried out for three-component PI- and PEI-composites under mild tribological loading conditions at a point contact (ball-on-disk, *P* = 5 N, *V* = 0.3 m/s) at room and elevated temperatures, despite the fact that the PTFE, Gr, and MoS_2_ solid lubricant mechanism was described in the literature [35]. To the best of our knowledge, this was not performed in a similar formulation before.

The paper aims to investigate the regularities of transfer film formation in high-strength anti-friction PEI- and PI-based composites in the temperature range of 23–180 °C (240 °C for PI). The second section describes materials and research methods. Section 3.1 presents data on the structure, physical and mechanical properties of the PEI-based composites. Section 3.2 is devoted to the analysis of tribological characteristics obtained according to the ‘ball-on-disk’ scheme on both metal and ceramic counterparts. Section 3.3 describes the results of elevated-temperature tribological tests of the PEI-based composites. In Section 3.4, a comparative analysis was carried out on the tribological properties of both three components PEI- and PI-based composites at the temperature range from 23 up to 240 °C. Section 4 focuses on the comparison of obtained results with the data described in the relevant literature. In conclusions, the mentioned data are summarized, and some practical recommendations are proposed on their preferred operating conditions.

## 2. Materials and Methods

### 2.1. Materials

In this study, both PEI and PI powders were purchased from the same supplier (Solver, Jiande, China). They had the same average particle size of 16 µm. The following commercially available fillers were utilized: the ‘Fluralit’ fine powder with an average diameter of less than 3 μm obtained by the F-4 fluoroplastic thermal decomposition (‘Fluralit synthesis’ LLC, Moscow, Russia); molybdenum disulfide MoS_2_ (Climax Molybdenum, Leadville, CO, USA, Ø 1–7 μm), as well as colloidal Gr (Ø 1–4 μm). Chopped Carbon Fibers (CCF, Tenax^®^-A, Teijin Carbon Europe Gmbh, Wuppertal, Germany) with lengths of 2 mm (an aspect ratio of about 100) were used for the reinforcement.

### 2.2. Fabrication of the Composites

**Figure 1:**
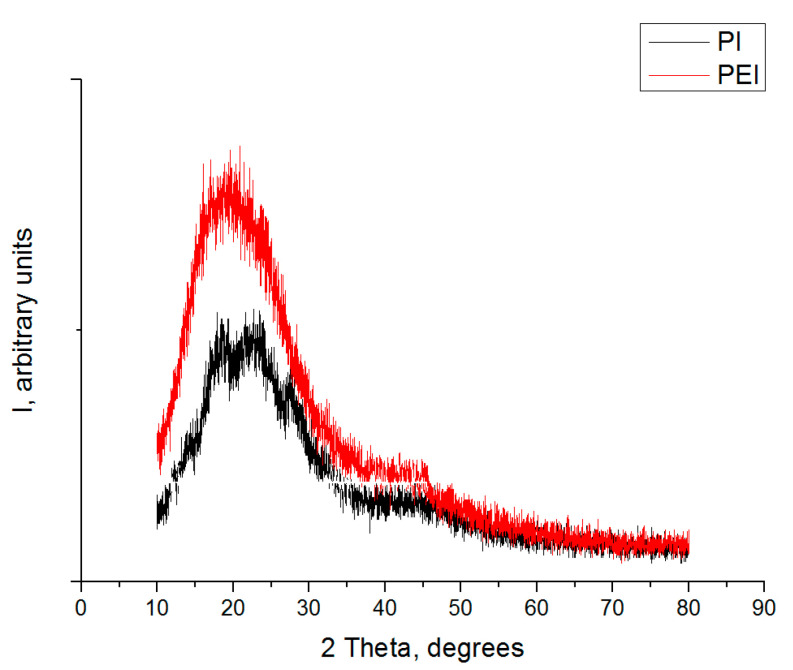
The X-ray diffraction patterns of the initial PEI and PI powders.

The polymer powders and the fillers were mixed by dispersing the suspension components in alcohol using a ‘PSB-Gals 1335-05′ ultrasonic cleaner (‘PSB-Gals’ Ultrasonic equipment center, Moscow, Russia). Processing duration was 3 min; generator frequency was 22 kHz. After mixing, a suspension of the components was dried in an oven with forced ventilation for 3 h at a temperature of 120 °C. The use of alcohol as a mixing medium suggested the absence of volatiles. Both PEI- and PI-based composites were fabricated by hot pressing at a pressure of 15 MPa and a temperature of 370 °C with a subsequent cooling rate of 2 °C/min. X-ray diffraction patterns and crystallinity degrees of the initial PEI and PI powders (before hot pressing) are presented in Figure 1 and Table 1.

Similar to the earlier studies [34], contents of CCF and all three types of the solid lubricant fillers were chosen to be 10 wt.%. In addition, ternary PEI- and PI-based composites were also fabricated containing 23 wt.% MoS_2_, which was identical to the volume content of the other two types of particles, since the MoS_2_ density is more than twice that of both Gr and PTFE. Compositions and the glass transition temperatures of the investigated samples are shown in Table 2.

### 2.3. Physical and Mechanical Properties

Tensile properties of the ‘dog-bone’ shaped PEI-based samples were measured using an ‘Instron 5582′ electromechanical testing machine (Instron, Norwood, MA, USA). The number of samples of each type was at least four. The following samples’ dimensions were taken: T = 3.2 ± 0.4 mm; W = 3.18 ± 0.5 mm; L = 9.53 ± 0.5 mm; WO = 9.53 + 3.18 mm; LO = 63.5 ± 0.4 mm; D = 25.4 ± 5 mm; R = 12.7 ± 1 mm. The strain was measured with an extensometer.

### 2.4. Tribological Characteristics

Dry sliding friction tests were carried out according to the ‘ball-on-disk’ scheme at a load (*P*) of 5 N and a sliding speed (*V*) of 0.3 m/s. A ‘THT-S-BE-0000′tribometer (CSEM, Neuchâtel, Switzerland) was used in accordance with ASTM G99. The maximum Hertzian contact pressure (*P_max_*) was 417 MPa. The tribological tests were conducted using metal and ceramic counterparts (balls made of bearing steel (60 HRC) and Al_2_O_3_, respectively). For both counterparts, their diameters were 6 mm, and the surface roughness was R*_a_* = 0.02 µm (it was assessed with the help of a ‘New View 6200′ optical interferential profilometer, ‘Zygo Corporation’, Middlefield, CT, USA). The testing distance was 1 km and the tribological track radius was 16 mm.

WR levels were determined by measuring width and depth of wear tracks according to stylus profilometry, followed by multiplication by their length. They were calculated taking into account both load and distance values:

(1) $$\mathrm{Wear}\;\mathrm{rate}\;=\frac{\mathrm{volume}\;\mathrm{loss}\;\left({\mathrm{mm}}^{3}\right)}{\mathrm{load}\;\left(N\right)\times \mathrm{sliding}\;\mathrm{distance}\;\left(m\right)}$$

### 2.5. Structural Studies

Surface topography of the wear tracks was studied using a ‘Neophot 2′ optical microscope (Carl Zeiss, Jena, Germany) equipped with a ‘Canon EOS 550D’ digital camera (Canon Inc., Tokyo, Japan), and an ‘Alpha-Step IQ’ contact profiler (KLA-Tencor, Milpitas, CA, USA).

The structural studies were carried out on cleaved surfaces of mechanically fractured notched specimens. To do so, liquid nitrogen was employed to cool them down. A ‘LEO EVO 50′ scanning electron microscope (Carl Zeiss, Oberkochen, Germany) with an ‘Oxford INCA X-Max80′ attachment for EDS analysis (Oxford Instruments, Abingdon, Oxfordshire, UK) was employed at an accelerating voltage of 20 kV.

Both phase composition and structural parameters were investigated using an ‘XRD-6000′ diffractometer with CuK_α_ radiation. The phase composition analysis was carried out by the ‘POWDER CELL 2.4′ software package with the ‘PDF 4+’ databases.

Glass transition temperature (T_g_) was determined using a SDT Q600 combined analyzer (TA Instruments, New Castle, DE, USA).

Glass transition temperature (Tg) was measured through heat capacity variation at polymer transition from glassy to highly elastic state. The scanning differential calorimetry (SDC) was employed with the use of an SDT Q600 combined analyzer (TA Instruments, New Castle, DE, USA). Heating rate was equal to 10 °C/min. 

## 3. Results and Discussion

This section presents the results of both mechanical and tribological tests of the PEI-based composites loaded with the reinforcing and solid lubricant fillers identical to those for the PI-based ones [34]. The temperature (T) range of the tribological tests was 23–180 °C.

### 3.1. The Structure, the Physical and Mechanical Properties of the PEI-Based Composites

**Figure 2:**
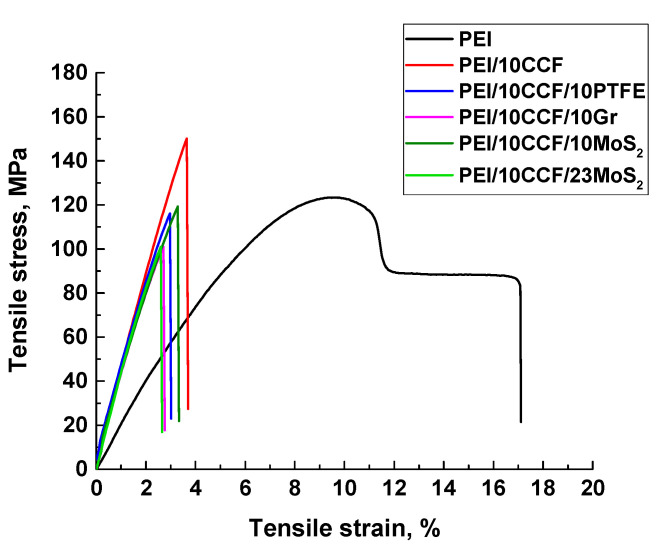
The stress–strain diagrams for neat PEI, as well as the PEI/10CCF, PEI/10CCF/10PTFE, PEI/10CCF/10Gr, PEI/10CCF/10MoS_2_, and PEI/10CCF/23MoS_2_ composites.

According to the data presented in Table 3 and Figure 2, the key mechanical properties of the PEI-based composites increased markedly after loading with 10 wt.% CCF: the elastic modulus values doubled, and the ultimate tensile strength levels increased by 1.2 times. The subsequent loading of the PEI/10CCF composite with solid lubricant particles slightly reduced their deformation-strength characteristics.

Note that the mechanical properties of neat PEI (elastic modulus, ultimate tensile strength, and elongation at break) exceeded the corresponding ones of neat PI by ~10%. This fact, among the others, was associated with the increased PEI crystallinity degree (32 vs. 21%). For the composites based on both polymers, the difference in the mechanical properties was offset by the CCF reinforcing effect. For all the studied ones, the elongation at break values decreased by 4–5 times, and their fracture had a brittle character.

**Figure 3:**
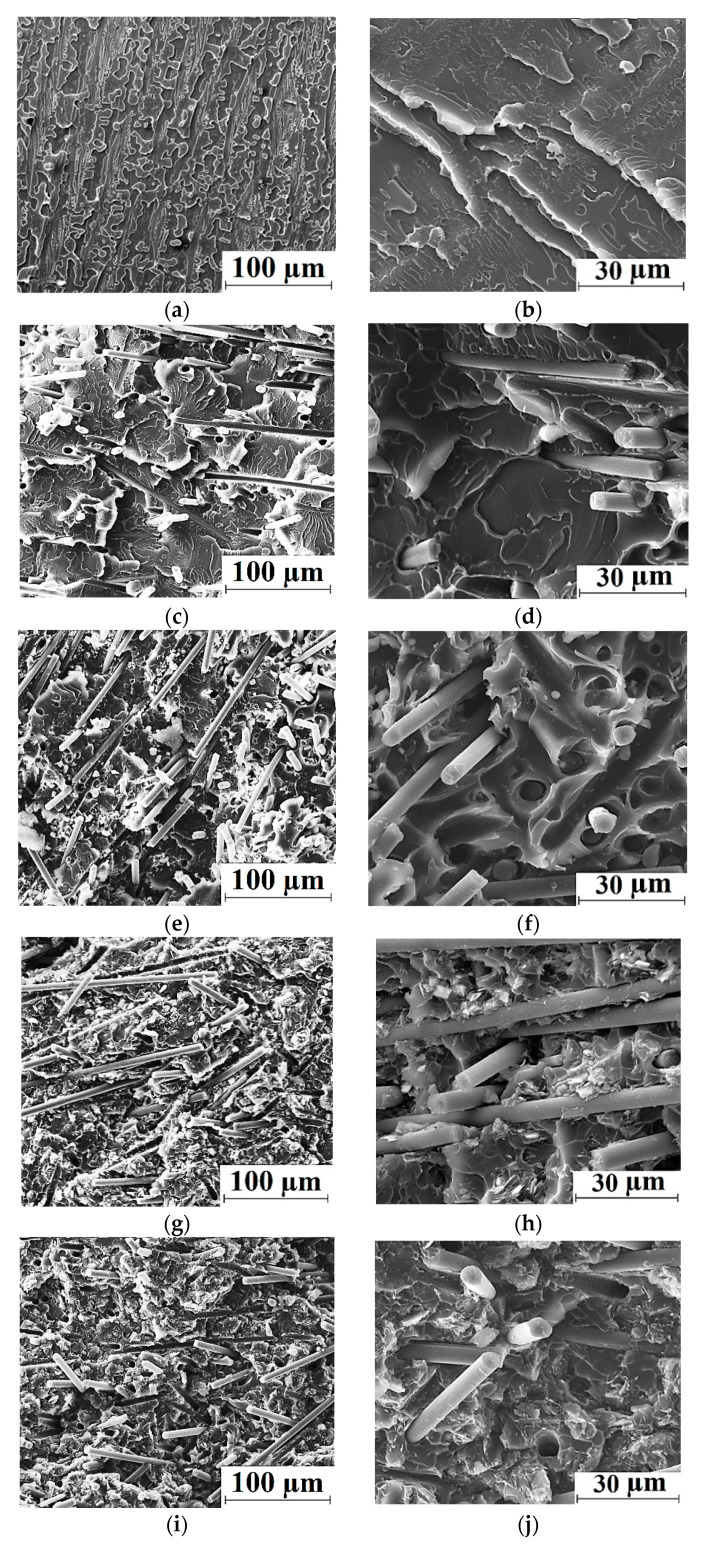
SEM-micrographs illustrating the structure for neat PEI (**a**,**b**), as well as the PEI/10CCF (**c**,**d**), PEI/10CCF/10PTFE (**e**,**f**), PEI/10CCF/10MoS_2_ (**g**,**h**), PEI/10CCF/10Gr (**i**,**j**) composites.

Figure 3 shows SEM micrographs on the structure of neat PEI and the PEI-based composites. The supermolecular structure of neat PEI (Figure 3a,b) was uniform; however, the “cell”-like shape of structural elements being characteristic of neat PI [34] was not evident. After loading with CCF, these reinforcing inclusions were distributed quite evenly over the bulk composite (Figure 3c,d). The solid lubricant fillers (PTFE, Gr and MoS_2_) were located uniformly as well; no visible signs of their agglomeration in the ternary composites were revealed (Figure 3e–j). Note that the adhesion of CCF to the polymer matrix was rather great (Figure 3h) after loading with all three types of solid lubricant particles. Thus, the mechanical properties of the ternary PEI/CCF-based composites were close to those for the similar PI/CCF-based ones [34], which correlated with the conformity of their molecular structures.

### 3.2. The Tribological Properties of the PEI-Based Composites

Table 4 presents both CoF and WR values for all the studied samples after the tests on both metal and ceramic counterparts. It could be concluded that loading with all types of the fillers caused a decrease in the CoF levels. The maximum effect was manifested for the PTFE-containing composite, whose WR value was reduced by 850 times in the metal-polymer contact and by 1400 times in the ceramic-polymer one. The inorganic solid lubricant fillers (Gr and MoS_2_) decreased the WR by ~27 (Gr) and 69 (MoS_2_) times for the metal-polymer interface and by ~90 times for the ceramic-polymer one compared to that of neat PEI.

**Figure 4:**
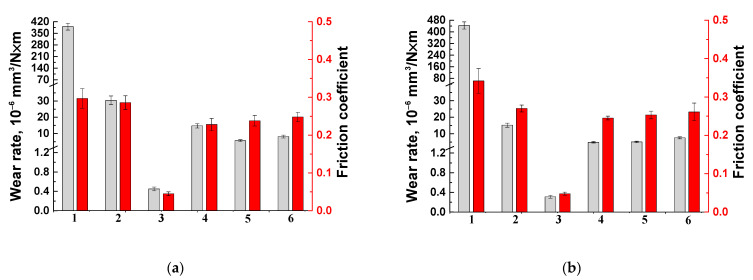
Wear rates and the coefficients of friction for neat PEI (1), as well as the PEI/10CCF (2), PEI/10CCF/10PTFE (3), PEI/10CCF/10Gr (4), PEI/10CCF/10MoS_2_ (5), PEI/10CCF/23MoS_2_ (6) composites. The metal (**a**) and ceramic (**b**) counterparts.

Figure 4 presents both WR and CoF bar-diagrams of all the studied composites for both metal- and ceramic-polymer tribological contacts. The composite loaded with PTFE showed the lowest (close to wearless mode) values in both cases.

For the PEI/10CCF-based composites loaded with Gr and MoS_2_ inorganic particles, the WRs decreased by two to three times depending on the counterpart material, while their CoF values differed slightly for both fillers. Thus, PTFE showed the maximum efficiency in terms of improving the tribological characteristics. According to the authors, this fact was associated with the formation of transfer films primarily on the sliding (wear track) surfaces in both metal- and ceramic-polymer tribological matings. For this reason, an analysis of the counterpart surfaces and the wear tracks on all the studied PEI-based composites was carried out.

**Figure 5:**
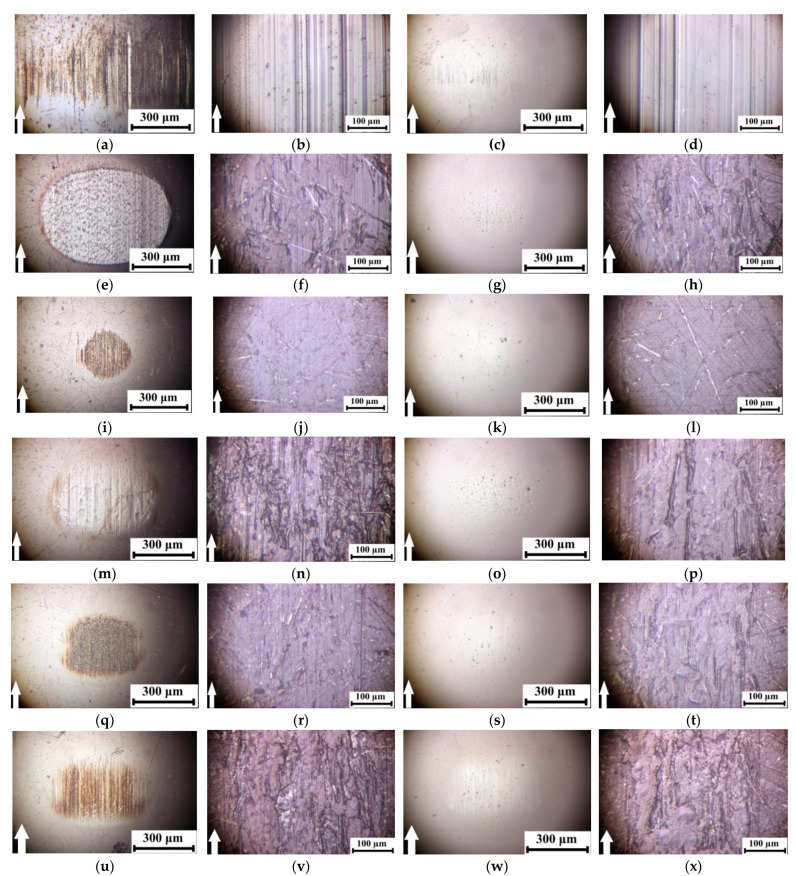
Optical micrographs of the metal counterpart surfaces and the friction tracks on neat PEI (**a**–**d**), as well as the PEI/10CCF (**e**–**h**), PEI/10CCF/10PTFE (**i**–**l**), PEI/10CCF/10Gr (**m**–**p**), PEI/10CCF/10MoS_2_ (**q**–**t**), and PEI/10CCF/23MoS_2_ (**u**–**x**) composites.

Figure 5 shows optical micrographs of the counterpart surfaces and the wear tracks on neat PEI and the studied composites. Under the friction of neat PEI on the steel counterpart, wide wear scars were evident with partially adhered debris on the counterpart surface under intense wear conditions (Figure 5a,b). According to the authors, this effect was associated with the rigid polymer that did not allow easy sliding of the smooth steel ball over its surface. As a result, high CoF and WR values were registered. A similar topology of the friction surface was typical for the ceramic counterpart (Figure 5d). In this case, debris was almost absent on the counterpart surface (Figure 5c).

Loading with CCF caused a fold decrease in the WR (Figure 4), although the CoF value reduced slightly. The CCF abrasive impact on the steel counterpart was observed, which, among other things, changed the contact pressure in the friction zone (Figure 5e). For obvious reasons, the ceramic counterpart was not worn out (Figure 5g). In both cases, there were no longitudinal grooves on the wear track surfaces due to the CCF hardening effect (Figure 5f,h).

Filling the binary PEI/10CCF composite with PTFE particles was accompanied by a sharp decrease in both WR and CoF levels (Figure 4). The composite wear track surface was smooth; only some scratches were visible as a result of the surface preparation with sandpaper before the tribological tests (Figure 5j,l). On the steel counterpart surface, a thin transfer film was found, width corresponded to that of the wear track (Figure 5i). Since a transfer film was hardly adhered on the ceramic counterpart surface, it was not revealed at all (Figure 5k).

After loading the PEI/10CCF composite with the inorganic Gr and MoS_2_ solid lubricant fillers (at both filling degrees), the CoF and WR values were slightly lower than those of the PEI/10CCF sample.

When Gr was loaded, fragments of debris were adhered to the composite friction surface in the form of patches, which were more pronounced for the steel-polymer tribological contact (Figure 5n,p). Debris was also attached to both counterpart surfaces, but this was mostly exhibited on the steel ball again (Figure 5m,o). On the metal counterpart surface, a thick transfer film was damaged by longitudinal grooves caused by the CCF scratching.

When filling with 10 wt.% MoS_2_, the composite wear track surface was smoother, and the wear track width was slightly thinner (Figure 5r,t). This corresponded to the transfer film dimensions on the steel counterpart surface (Figure 5q) that was practically not detected in the second case (Figure 5s). Increasing the filler content up to 23 wt.% almost did not change both the CoF and WR values (Figure 4). However, the friction surface topography was more reminiscent of that on the ternary composite loaded with Gr, where some fixed fragments of debris were found (Figure 5v,x). At the same time, both the area and visually estimated thickness of the transfer film were greater on the metal counterpart surface (Figure 5u,w).

Thereby, the similarity of the surface topology patterns on the wear tracks and the WR levels of the ternary PEI-based composites with different counterpart materials indicated the predominant influence of the transfer film formation on the wear process. In this case, the role of the solid lubricant filler was manifested in two ways: (a) the ability to form an anti-friction film due to layer-by-layer fracture (peeling) of such particles, and (b) the ability to adhere this film to the composite surface (including due to the development of oxidative processes in the tribological contact, which could be facilitated by the presence of oxygen atoms in PEI macromolecules). According to the authors, such an anti-friction film was formed on the ternary PEI-based composite loaded with PTFE. When filling with Gr and MoS_2_ particles, they acted as dispersed hardening inclusions, for which the applied load level was insufficient for delamination in the form of flakes as typical solid lubricants.

**Figure 6:**
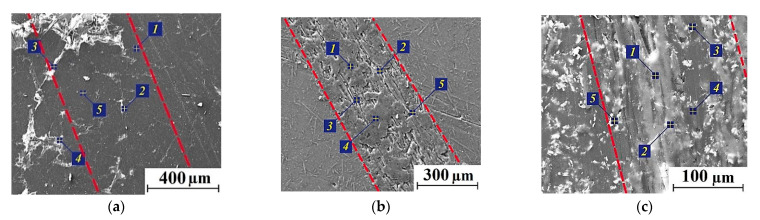
SEM-micrographs of the wear tracks on the PEI/10CCF/10PTFE (**a**), PEI/10CCF/10Gr (**b**), PEI/10CCF/10MoS_2_ (**c**) composites; *T* = 23 °C (wear track edges marked with red dotted lines).

As part of the substantiation of this assumption, below are the results of EDS analysis of the composite wear track surfaces after sliding in the ceramic-polymer contacts (Figure 6 and Table 5). In addition, this aspect is discussed below for high temperature conditions of the tribological tests.

The results presented in Figure 5 and Figure 6, as well as Table 5, testified in favor of the formation of thin homogeneous anti-friction PTFE-containing films on the wear track surfaces (at spectrum points 3, 4, and 5, the fluorine content exceeded 40 at.%). For the MoS_2_ case, both molybdenum and sulfur contents were several times lower. Figure 6b presents the entire wear track width of the ternary PEI-based composite loaded with Gr particles where the fragmentary debris adherence was evident. The C content was almost the same both in the fixed debris fragments and in ‘dimples’, where CCF protrusions were found. In addition, an inhomogeneous transfer film was observed on the ternary composite surface filled with MoS_2_ (Figure 6c), but it was not as fragmented. However, MoS_2_ particles did not ensure formation of any uniform films.

Since thermoplastic PEI is a high-temperature polymer, in order to establish the role of the solid lubricant fillers at elevated temperatures, additional tribological studies were carried out. First of all, the authors analyzed the evolution of transfer films with an increase in the test temperature and their influence on changes in both CoF and WR values. Due to the fact that the counterpart materials did not exert a significant effect on the tribological characteristics of the composites (Figure 4), the only ceramic one was used to minimize the impact of oxidative processes.

### 3.3. High Temperature Tribological Tests of the PEI-Based Composites

Table 6 presents the results of the high-temperature tribological tests of the investigated PEI-based composites in the ceramic-polymer contact. The CoF values increased with rising the test temperature, excluding both PEI/10CCF/10MoS_2_ and PEI/10CCF/23MoS_2_ samples, for which the CoF level decreased by several times at *T* = 180 °C compared to those (CoF ≈ 0.10–0.11) at room temperature. On the other hand, a low CoF value was characteristic of the PEI/10CCF/10PTFE composite, despite the fact that it doubled (CoF~0.05–0.11) as the temperature rose from 23 to 180 °C.

At the high test temperatures, the WR values differed with the filler type. For the composite loaded with PTFE, low WR levels of (0.20–0.31) × 10^−6^ mm^3^/N m were observed over the entire temperature range. Loading with Gr multiplied the WR values as the test temperature increased. The presence of MoS_2_ particles initially enhanced the WR levels as the temperature increased up to 120 °C, but then sharply reduced at *T* = 180 °C to a level commensurate with that at room temperature. An increase in the MoS_2_ content up to 23 wt.% contributed to a decrease in the WR values by several times at both elevated temperatures (120 and 180 °C).

**Figure 7:**
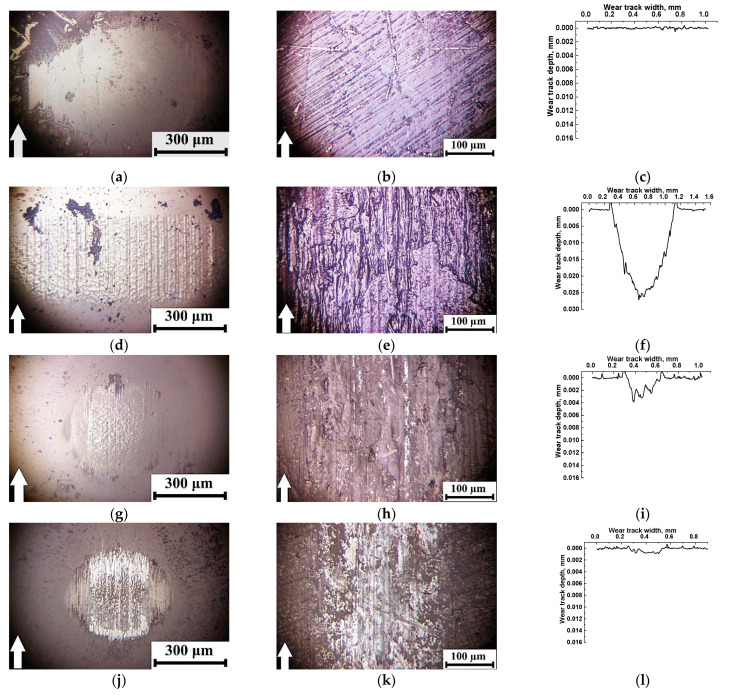
Optical micrographs of the friction scar surfaces on the ceramic counterpart (*T* = 180 °C) after the tribological tests of the PEI/10CCF/10PTFE (**a**–**c**), PEI/10CCF/10Gr (**d**–**f**), PEI/10CCF/10MoS_2_ (**g**–**i**), PEI/10CCF/23MoS_2_ (**j**–**l**) composites.

In order to understand the reason for the observed phenomena, an analysis of the counterpart surfaces and the friction tracks of the ternary PEI-based composites was carried out (Figure 7).

The friction of the PEI/10CCF/10PTFE composite developed almost in the wearless mode (Figure 7c). In Figure 7b, scratches were found due to grinding the surface with sandpaper (during specimen preparation). As shown below, an anti-friction PTFE film on the wear track surface provided an extremely low CoF level of 0.048. Only debris fragments in the form of dark patches were evident on the counterpart surface (Figure 7a). At the same time, the transfer film formed on the composite wear track surface was stably preserved over the entire studied temperature range of 23–180 °C.

After intense wearing, the wear track depth was up to 0.033 mm and agglomerates of debris particles were adhered to the surface of the PEI/10CCF/10Gr composite. On the counterpart surface, a large film of attached debris particles was found that was damaged by numerous longitudinal grooves (Figure 7d,e).

WR levels were lower for the PEI/10CCF/10MoS_2_ composite when compared to the previous case. At the low MoS_2_ content of 10 wt.%, a transfer film on the wear track surface was more fragmented, and a similar one, formed from debris particles, was evident on the ceramic counterpart surface. With an increase in the MoS_2_ content up to 23 wt.%, the transfer film on the composite wear track surface was more solid (Figure 7k), which corresponded to both CoF lowering (down to 0.1) and WR rising. At the temperature of 180 °C, debris adhered to the counterpart surface and formed an appropriate film. It is suggested that enhancing the test temperature stimulated the development of oxidative processes, contributed to the adhering of the transfer film on the counterpart surface and the transformation of the friction mechanism into the ‘polymer-on-polymer’ mode (Figure 7j).

**Figure 8:**
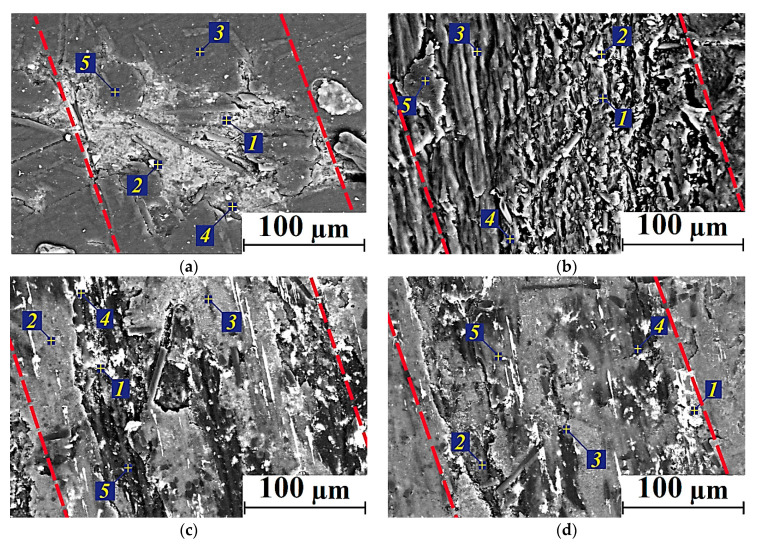
SEM-micrographs of the wear tracks on the PEI/10CCF/10PTFE (**a**), PEI/10CCF/10Gr (**b**), PEI/10CCF/10MoS_2_ (**c**), PEI/10CCF/23MoS_2_ (**d**) composites after the tribological tests at *T* = 180 °C (wear track edges marked with red dotted lines).

The results of EDS analysis of the transfer films formed on the composite wear track surfaces during the tests at *T* = 180 °C are presented in Figure 8 and Table 7. One of these films was fixed on the wear track surface of the PEI/10CCF/10PTFE composite (Figure 8a). However, the average CoF value doubled at the high test temperature of 180 °C relative to that at *T* = 23 °C. According to the authors, this was mainly due to an increase in the CoF level of the polymer itself with rising the test temperature.

On the PEI/10CCF/10Gr composite, no continuous anti-friction Gr-containing transfer film was formed (Figure 8b). As a result, the CoF level increased significantly up to 0.45, which was almost twice as high as at *T* = 23 °C. The absence of a protective transfer film was confirmed by the data of EDS analysis, which were approximately the same at all examined points (Table 7).

At the high test temperature of 180 °C, the presence of MoS_2_ particles at the greatest of the studied concentrations (23 wt.%) contributed to the formation and adherence of a more continuous and stable transfer film on the wear track surface of the PEI/10CCF/10MoS_2_ composite due to oxidative processes in the tribological contact. The results of EDS analysis expectedly showed an increase in both Mo and S contents on the wear track surface (Figure 8d). At the lower MoS_2_ content of 10 wt.%, the transfer film on the composite wear track surface was not continuous, but fragmented (Figure 8c).

The interpretation of the obtained results was carried out in terms of adhering the transfer films to the composite wear track surfaces, which significantly depended on the nature of the polymer matrix [36]. For this reason, a comparison of the tribological characteristics at elevated temperatures is reported below for the ternary polymer/CCF/solid lubricant filler composites with different matrix materials, namely PEI and PI.

### 3.4. The Results of the Comparative Analysis of the High-Temperature Tribological Properties of the PEI- and PI-Based Composites

Table 8 shows both CoF and WR tribological characteristics for the PI-based composites at various temperatures. Since these samples had the higher glass transition temperatures of about 260 °C (according to Table 2) compared to those for the PEI-based ones, the temperature range of the tribological tests was extended up to 23–240 °C. Table 8 shows that both CoF and WR levels increased with the rising tribological test temperature for all the composites except the PEI/10CCF/23MoS_2_ one. Note that, in contrast to the ternary PEI-based composites, the presence of PTFE enabled us to maintain the low WR values only up to 120 °C, after which they increased by many times (WR = 72 and 68 × 10^−6^ mm^3^/N m at *T* = 180 and 240 °C, respectively). According to the hypothesis developed in this study, the reason was the impossibility of retaining a PTFE-containing film on the composite sliding (wear track) surfaces. On the other hand, loading the composites with 23 wt.% MoS_2_, in contrast to the PEI-based samples, resulted in a noticeable decrease in both CoF (down to 0.08) and WR (down to 2.8 × 10^−6^ mm^3^/N m) levels already at *T* = 120 °C. This trend continued up to the highest tribological test temperature.

**Figure 9:**
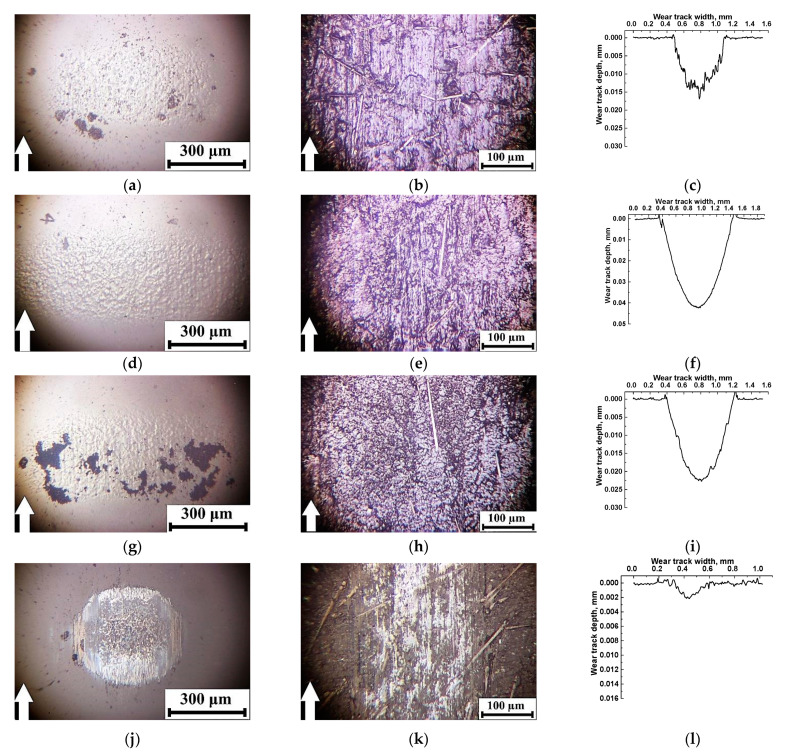
Optical micrographs of the friction scar surfaces on the ceramic counterpart after the tests of the PI/10CCF/10PTFE (**a**–**c**), PI/10CCF/10Gr (**d**–**f**), PI/10CCF/10MoS_2_ (**g**–**i**), PI/10CCF/23MoS_2_ (**j**–**l**) composites at the temperature of 180 °C.

Figure 9 presents optical micrographs of the counterpart surfaces and the wear tracks on the PI-based composites after the tests at *T* = 180 °C. Intensive wear of the ternary composites containing only 10 wt.% of solid lubricant particles was accompanied by the formation of transfer films on the ceramic counterpart surfaces, not damaged with transverse grooves (Figure 9a,d,g). Simultaneously, adhered debris fragments were evident on the composite wear track surfaces (Figure 9b,e,h). Only in the case of the MoS_2_ content of 23 wt.%, the formation and adherence of a MoS_2_-containing transfer film were ensured on the composite wear track surface (Figure 9k), but it was not continuous. Traces of this solid lubricant filler were fixed on the surface of the ceramic counterpart as well (Figure 9j).

**Figure 10:**
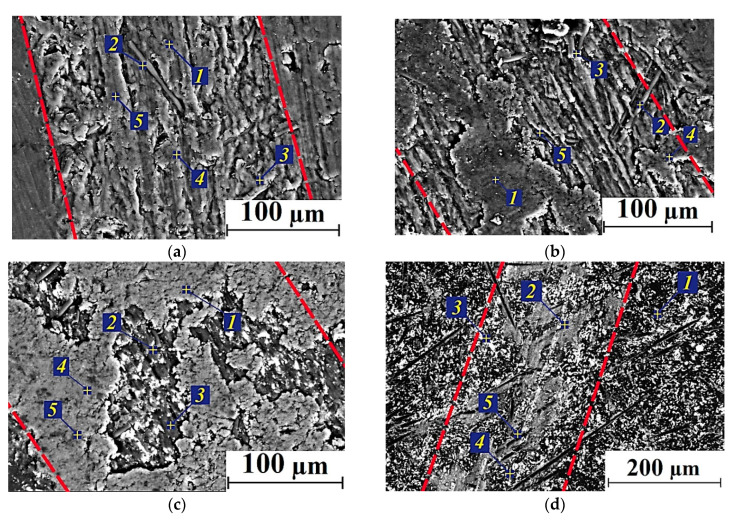
SEM-micrographs of the wear track surfaces on the PI/10CCF/10PTFE (**a**), PI/10CCF/10Gr (**b**), PI/10CCF/10MoS_2_ (**c**), PI/10CCF/23MoS_2_ (**d**) composites after the tests at the temperature of 180 °C (wear track edges marked with red dotted lines).

SEM-micrographs of the wear tracks on the ternary composites after the tribological tests at *T* = 180 °C and the results of their EDS analysis are presented in Figure 10 and Table 9, respectively. On the wear track surfaces of the samples containing only 10 wt.% solid lubricant particles, transfer films were fragmentary, while it was more continuous at 23 wt.% MoS_2_. For the PI/10CCF/10PTFE composite, the data of EDS analysis (Table 9) indicated a lower F content in the transfer film compared to the PEI/10CCF/10PTFE sample (Table 7). In both PI/10CCF/10MoS_2_ and PI/10CCF/23MoS_2_ cases, appreciable amounts of oxygen were found in addition to the expected Mo and S elements (Table 9). This indicated the intensive development of oxidative processes, including those contributing to the adhering of the transfer films on the composite wear track surfaces.

**Figure 11:**
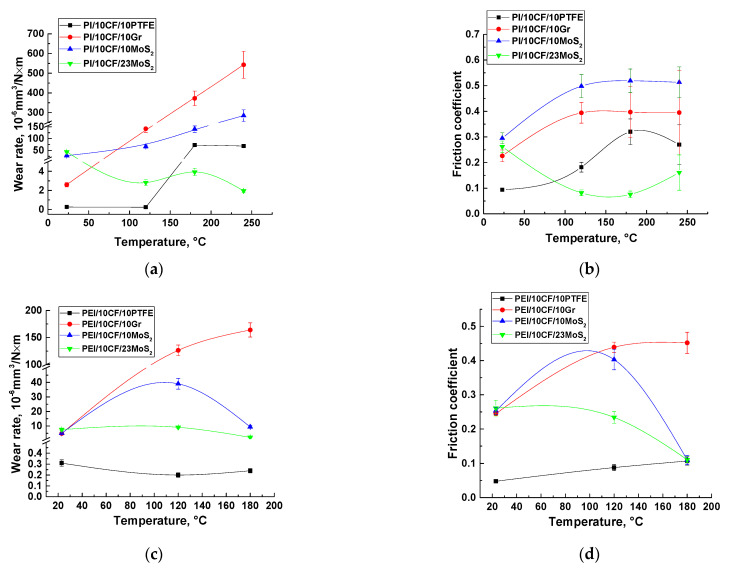
Wear rates (**a**,**c**) and the coefficients of friction (**b**,**d**) for the PEI- and PI-based composites. The ceramic counterpart.

The comparative analysis of both WR and CoF versus temperature dependences of the ternary PEI- and PI-based composites (Figure 11) enabled us to draw the following conclusions:

- For the PEI-based samples loaded with PTFE, the wearless mode (WR < 0.3 × 10^−6^ mm^3^/N m) was realized at low CoF levels (≤0.1) in the entire studied temperature range (*T* = 23–180 °C);
- For the PI-based ones identical in filling, PTFE ceased to play the role of a solid lubricant filler already at *T* = 180 °C, which corresponded to a sharp increase in CoF values (>0.3);
- For the PEI-based composites containing the lower MoS_2_ amount of 10 wt.%, these particles contributed to the solid lubricant mode only when the test temperature rose up to 180 °C, which corresponded to reducing the CoF values down to ~0.1;
- Enhancing the MoS_2_ content up to 23 wt.% in the PEI-based composites provided stable WR levels of <10 × 10^−6^ mm^3^/N m, although the CoF values reached ~0.1 only at *T* = 180 °C;
- The twofold change in the MoS_2_ content (10 and 23 wt.%) in the PEI-based composites contributed to equally low CoF values of about 0.1 at *T* = 180 °C, while WR levels of <(2–6) × 10^−6^ mm^3^/N·m also remained comparably negligible;
- The high MoS_2_ content of 23 wt.% in the PI-based samples was sufficient for providing low CoF levels of ~0.1 at all the elevated temperatures of the tribological tests (*T* = 120–240 °C), while ensuring negligible WR values of <(2–4) × 10^−6^ mm^3^/N·m;
- Loading both PEI- and PI-based composites with MoS_2_ solid lubricant particles did not contribute to ultra-low WF levels even when CoF values of 0.1 were reached, similar to the PTFE-containing composites based on the same polymers.

In [34], the authors showed that both CoF and WR levels correlated well with each other when testing the PI/CF/solid lubricant filler composites according to the ‘ball-on-disk’ scheme. In this case, their values were determined by the formation of a transfer film on the composite wear track surfaces. Since the WR values were assessed at the end of the tribological tests, only the CoF versus time dependence (recorded in situ) could be a clear factor shedding light on the nature of the developing processes.

**Figure 12:**
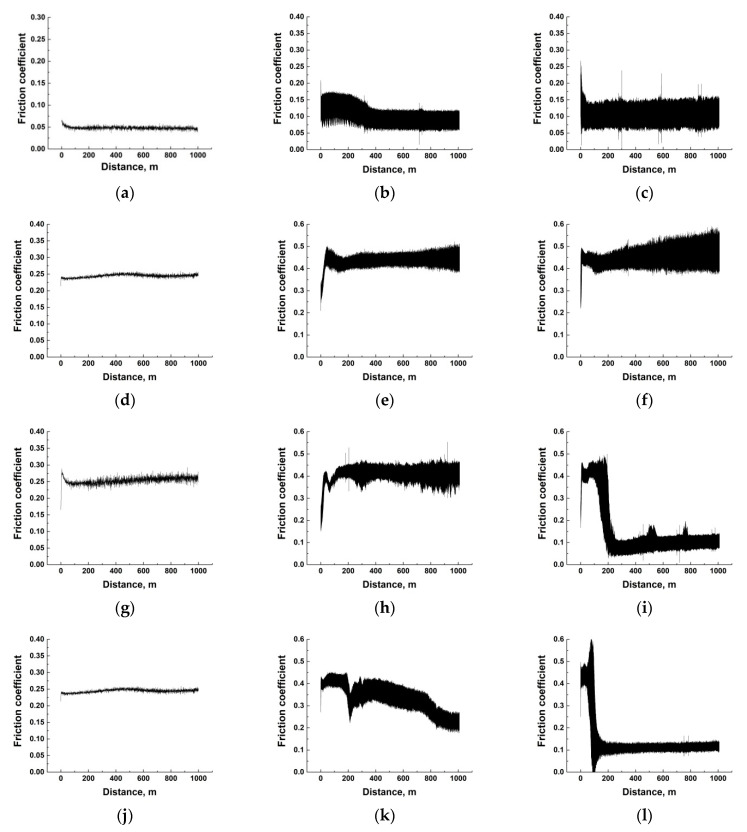
The dependences of CoF versus distance for the PEI/10CCF/10PTFE (**a**–**c**), PEI/10CCF/10Gr (**d**–**f**), PEI/10CCF/10MoS_2_ (**g**–**i**), PEI/10CCF/23MoS_2_ (**j**–**l**) composites. The test temperatures of 23 °C (**a**,**d**,**g**,**j**), 120 °C (**b**,**e**,**h**,**k**), and 180 °C (**c**,**f**,**i**,**l**).

**Figure 13:**
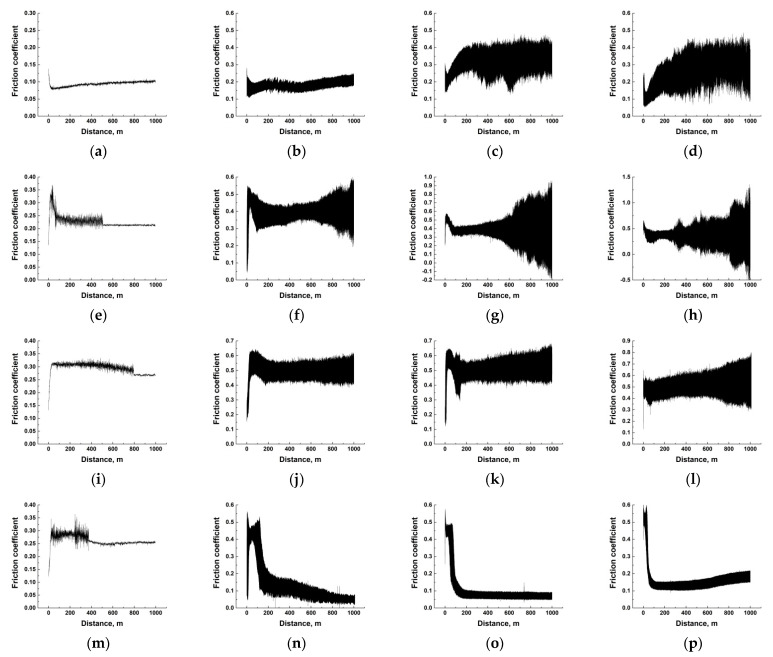
The dependences of CoF versus distance for the PI/10CCF/10PTFE (**a**–**d**), PI/10CCF/10Gr (**e**–**h**), PI/10CCF/10MoS_2_ (**i**–**l**), PI/10CCF/23MoS_2_ (**m**–**p**). The test temperatures of 23 °C (**a**,**e**,**i**,**m**), 120 °C (**b**,**f**,**j**,**n**), 180 °C (**c**,**g**,**k**,**o**), and 240 °C (**d**,**h**,**l**,**p**).

Figure 12 and Figure 13 show the ‘CoF versus distance’ dependencies for the composites at all the studied temperature range (*T* = 23–180 °C for the PEI-based samples and *T* = 23–240 °C for the PI-based ones). The general trend was rising in both CoF values and their oscillations with an increase in the test temperature. Since the composite structure, the counterpart material and its roughness did not change with increasing the temperature, the observed effect was due to the polymer matrix nature (which should manifest itself to different degrees for the PEI- and PI-based composites). Based on this assumption, interpretations of these dependences grouped by the solid lubricant filler types are given below.

***PTFE.*** In the PEI matrix case, the CoF values and their oscillations increased with rising temperature, but their average levels barely exceeded 0.11 (Figure 12a–c). For the similar PI-based composites, the CoF behavior was similar, but its mean level was greater than 0.3 at the maximum test temperature (Figure 13a–c). According to the authors, the rigid PI matrix was unable to adhere to a transfer film at such a high level of CoF oscillations, so the ceramic counterpart wore out the composite intensively.

***Graphite.*** This solid lubricant filler type basically did not execute the discussed function. Therefore, both the magnitude and the level of oscillation of the CoF values increased significantly with rising the test temperature. This manifested itself to a greater extent for the PI-based samples (Figure 13e–h). In the PEI-based composites, macromolecules of the matrix polymer were more flexible, so the CoF oscillations were markedly lower at the same temperatures in the tribological tests (Figure 12d–f).

***MoS_2_ (10 wt.%).*** With the low content of these solid lubricant particles in the PI-based composites, patterns of the changes in the CoF values with temperature generally resembled those for the PI-based samples loaded with Gr (Figure 13i–l) described above. For the PEI-based ones, the wear behavior only changed at the high temperature of 180 °C. At the same time, the CoF values were high initially (similar to the running-in stage that resulted in the relatively high final WR level). However, the low CoF value of about 0.1 provided friction in the solid-lubricant sliding mode (Figure 12i).

***MoS_2_ (23 wt.%).*** The high content of this filler type resulted in the inhomogeneous structure of this ternary composite and, to a certain extent, deteriorated its mechanical properties. However, this also provided decreasing levels of both CoF and WR at certain temperatures. An interesting, revealed feature was the constancy of the low level of CoF (~0.1) for the PI-based composites at *T* = 120–180 °C (Figure 13n–p), while the CoF values did not reach low CoF ≈ 0.1 for the similar PEI-based sample at *T* = 120 °C (Figure 12k). According to the authors, the conditions were more severe for the PI-based composite at *T* = 120 °C from the very beginning of the tribological test due to the extremely high CoF level of 0.5. This stimulated the development of oxidative processes during oscillation of the CoF values and could be the cause of the partial material oxidation on the composite friction surface, followed by the anti-friction transfer film adhering, which reduced the CoF level down to ≈0.1.

**Figure 14:**
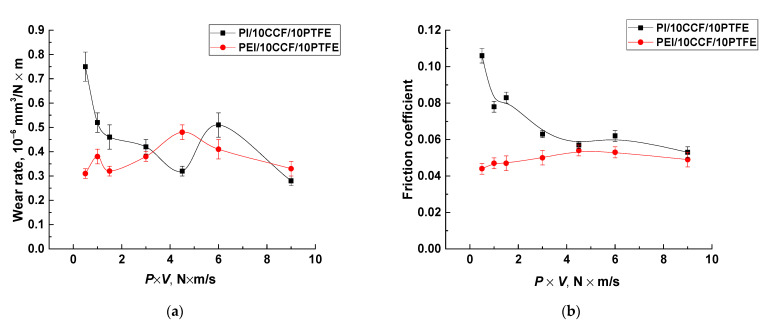
Wear rates (**a**) and the coefficients of friction (**b**) for the PI/10CCF/10PTFE and PEI/10CCF/10PTFE composites.

Since the authors appealed to the molecular structure of the polymer matrices when discussing the obtained results, the following experiment was very indicative. For the composites possessing the best tribological characteristics, namely the PEI/10CF/10PTFE and PI/10CF/10PTFE ones, the ‘rigidity’ of the tribological test conditions was varied by changing the (*P*·*V*) products at room temperature only. Figure 14 shows both WR and CoF versus (*P*·*V*) product dependences for the mentioned composites. For the PEI-based sample, the CoF and WR levels changed insignificantly throughout the studied range of loads and sliding speeds (*P* = 5–15 N, *V* = 0.1–0.6 m/s). In contrast, both CoF and WR values were higher for the PEI/10CF/10PTFE sample at low *P·V* values, but became close in magnitude at *P*·*V* = 9 N·m/s. This indirectly testified in favor of the greater pliability of the PEI matrix, since its role could be predominant under the ‘mild’ conditions. On the other hand, the role of reinforcing fibers increased under ‘severe’ conditions, and the materials functioned more in the ‘composite mode’. This could also characterize the ability of the anti-friction PTFE film to be retained on the composite wear track surface at great CoF oscillations.

The key principle of designing self-lubricating composites is addition of the fillers, which contributed to the formation of a transfer film (layer) onto friction surfaces through the development of thermal, mechanical and chemical processes. The main effective functioning condition is the long-term preservation of continuity and adhesion under the cyclic contact loads. A large number of studies and reviews were devoted to the analysis and systematization of such data [37,38,39,40,41,42,43,44,45]. In the present study, two types of solid lubricant fillers of different natures were used, namely crystalline, both Gr and MoS_2_, as well as thermoplastic, PTFE. The fundamental difference in their structures along with the physical, thermal and mechanical properties determined the variations of both the formation of the transfer films and their adhering on the wear track surfaces of the investigated composites (and on the counterpart surfaces as well).

The crystalline structure of Gr (as well as MoS_2_/WS_2_) possesses a hexagonal pattern. Lamellas are fixed by weak Van der Waals forces, providing low interlamellar strengths. Development of shearing force along the basal planes result in intercrystallite slips [46]. The nature of the Gr-induced lubrication is conventionally treated through adsorption due to ambient humidity. In doing so, vacant covalent bonds and edges of the basal planes are passivated. However, Gr is characterized by intense friction under dry sliding conditions. In contrast, unsaturated bonds on the basal plane edges react with oxygen and environmental moisture. Thus, MoS_2_-containg tribological oxidation products are formed [47].

As a numerical indicator, characterizing the difference between these two types of the crystalline fillers, the interlayer binding energy could be estimated. It is equal to 1.4 eV/atom for Gr, while it is much lower (0.056 eV/atom) for MoS_2_ [48,49,50].

The anti-friction nature of PTFE resulted from its molecular arrangement and low intermolecular cohesion. In contrast to both Gr and MoS_2_, there are no unsaturated bonds in PTFE. In this case, a thin transfer film can be formed at low contact stresses of about 10 MPa. The reason for the low CoF levels of about 0.1 is related to its easy formation process since the negligible intermolecular cohesion favored simple drawing of molecular chains out of crystalline regions. In addition, low thermal conductivity suppressed heat dissipation, which could be a reason for the transfer film failure because of local melting [46]. According to the authors, the formation of the transfer film on the friction surface of the triple composite loaded with PTFE was significantly facilitated in comparison with both Gr and MoS_2_.

Thus, the PTFE (polymeric) solid lubricant filler at a content of 10 wt.% was more efficient in terms of the formation of the stable transfer film on the wear track surface of the ternary PEI-based composite reinforced with 10 wt.% CCF in the entire studied ranges of temperatures (23–180 °C), loads (5–15 N) and sliding speeds (0.1–0.6 m/s), compared to the PI matrix. The MoS_2_ solid lubricant filler fixed the transfer film on the composite sliding surface due to the presence of oxygen atoms in PEI molecules, protecting it and providing high wear resistance of the PEI/10CCF/10MoS_2_ composite at the maximum test temperature of 180 °C. The most advanced composite was the PI/10CF/23MoS_2_ one in terms of improved high-temperature wear resistance (up to *T* = 240 °C), as well as its provision in the range *T* = 120–240 °C due to the consistently low CoF value and the formation of the transfer film on the wear track surface.

## 4. Discussion

It was frequently shown in the literature that the tribological characteristics of various materials were determined, not by their intrinsic properties, but by the interaction of the sliding parts of friction units, forming a complex self-organizing system [51]. In the cases of thermoplastic polymers or their composites, the ambient temperature, in general, and the tribological contact, in particular, exerted a significant effect on the system components [2]. The reason was the temperature dependence of the viscoelastic characteristics of such materials.

Moreover, the influence of the test temperatures on the TF formation process was fundamentally different for neat PI and its composites. In [52], an abrupt WR change was reported for a polyimide TF at temperatures both above and below that of the glass transition. Wear rate at temperatures above the transition could be up to 600 times less than that below the transition. Samyn et al. [53] revealed a transition in both CoF and WR in polyimide at 180°C. The obtained results were interpreted through different orientations of molecular conformation at the polymer sliding surface at *T* = 180 °C. Ma et al. found [54] that elevated temperature had a significant effect on the tribological properties of neat PI. In particular, its specific WR level was more than an order of magnitude fewer at T > 300 °C than that at lower test temperatures. This drastic improvement of the tribological properties was attributed to the easier transfer film formation at elevated temperatures. 

On the other hand, temperature rise affects tribological properties of PEI or PI-based composites in a different manner. For example, loading with conventional graphite flakes gradually exhibited poor adhesion to counterparts under elevated temperatures. The reason was the desorption of hydrocarbon.

At elevated temperatures, the tribological behavior of the PEI- and PI-based composites differed fundamentally depending on the loaded fillers. Xian et al. [32] studied PEI composites containing 5 vol.% nano-TiO_2_ or micro-CaSiO_3_. At room temperature, the samples filled with microparticles had the lowest WR, while the ones loaded with nano-TiO_2_ possessed the fewest WR at elevated temperatures.

Li et al. found [55] that the addition of sub-micro particles further reduced both CoF and WR values of the PEI-based composites, especially at elevated temperatures. Samyn, et al. showed [27] that the sliding mechanisms of PI/PTFE composites were determined by thermally controlled sliding of PTFE at temperatures above 120 °C, while polyimide degraded into a monomer above 180 °C. The lower friction induced by the presence of PTFE particles protected the PI subsurface layer through suppressing the reorientations of molecular structures. 

Dong et al. reported [56] that the presence of reinforcing CFs in PI-based composites protected them from intense wear and reduced CoF values at 180–260 °C due to improved lubricity. In addition, these authors found [57] that a PI-based composite containing 20 vol.% CF showed extremely low CoF levels of 0.054 and 0.1 at high temperatures of 180 and 260 °C, respectively, due to negligible WR values. However, these results were not consistent with those of Zhao et al. [58], according to which, the WR levels enhanced monotonically for PI-based composites reinforced with aramid, glass, and carbon fibers with an increase in temperature from room temperature up to 200 °C.

Duan et al. [59] evidenced that extremely low CoF and WR of thermosetting polyimide (TPI)-composite at high temperatures is attained at loading with aramid pulps (APs). In addition, an obvious decrease in wear rate and CoF in “TPI-aramid pulps” composites was registered under 200 °C. Combined loading of the TPI with graphite, g-C3N4 and the APs ensured simultaneous improvement in load-carrying capability and durability of tribofilms. A relevant study of Samyn et al. [60] focused on high-temperature wear resistance of “PI-CF”-composite loaded with solid internal lubricant (PTFE) or silicon oil at temperatures up to 260 °C.

In addition to the type and amount of fillers, as well as the test temperature, many factors affected the CoF and WR values. However, the results were actually determined by the regularities of the TF formation, adhering and degradation [35]. Among the key influencing factors, the following should be noted: the sliding speed, normal load, environmental temperature, surface roughness, counterpart material, polymer structure, types of both fillers and lubricating medium (if any), etc. [61,62,63,64,65].

The cited data are summarized in Table 10, including a comparison with the results obtained in these studies.

The authors considered the following statements as the key novelty of the obtained results. Firstly, the tribological test conditions were mild: the smooth ceramic (chemically resistant) counterpart slid over the surfaces of the fiber-reinforced HPP-based composites according to the ball-on-disk scheme at low *P·V* values. In addition, loading with PTFE solid lubricant particles, along with the strengthening effect of CCF, facilitated the formation and adherence of the TF on the sliding surfaces of the more flexible PEI-based composite at room temperature. In this case, a very low coefficient of friction (CoF) value of about 0.05 was observed. For the more rigid identically filled PI-based composite, the CoF value was twice as high under the same conditions. At elevated temperatures, rising both CoF levels and oscillation of their values made it difficult to retain the non-polar PTFE transfer film on the sliding surfaces of the PI-based composite. As a result, friction of the ceramic counterpart proceeded over the composite surface without any protecting TF at *T* ≥ 180 °C. For the sample with the more flexible PEI matrix, the PTFE-containing TF was retained on its sliding surface, providing a low WR level even under CoF rising and oscillating conditions.

Crystalline MoS_2_ was the less effective solid lubricant filler. Even with the TF formation and adherence on the sliding surface of the PEI- and PI-based composites and the low CoF values of ~0.1 (at elevated temperatures), the WR levels were an order of magnitude higher than those after loading with PTFE (WR > 2 × 10^−6^ mm^3^/N m). This was due to the greater energy consumption for the separation of flakes of this laminar filler [67]. At the same time, the material of the polymer matrix had an excellent effect on the TF formation and fixation. For the PI-based composite, the high CoF value at the initial friction stage stimulated the TF formation, which sharply reduced the CoF value down to 0.1 already at *T* = 120 °C. Rising the test temperature only contributed to the TF adherence on the composite sliding surface via the activation of oxidation processes. For the composite based on the more flexible PEI matrix, the intensity of deformation processes was not sufficient for the TF formation at *T* = 120 °C. At the same time, the CoF levels gradually decreased from 0.40 down to 0.25 (at the end of the tests). At *T* = 180 °C, the TF formed quite quickly due to the increase in the CoF level up to 0.45–0.50 at the initial testing stage, which caused the sharp decrease in the CoF value down to 0.1, similarly to the identically filled PI-based composite.

## 5. Conclusions

The structure, the mechanical and tribological properties of the PEI- and PI-based composites reinforced with CCF and loaded with solid lubricant fillers of various nature/structure (PTFE, Gr and MoS_2_) were studied in the metal- and ceramic-polymer tribological contacts in the temperature range of 23–180 (240) °C. It was shown that loading with 10 wt.% CCF 2 mm long doubled the elastic modulus value and enhanced the ultimate tensile strength level by 1.2 times. However, the mechanical and tribological properties of these ternary composites correlated to a small extent, which was associated with the determining role of the transfer films on their wear track surfaces. The effect of the different stiffnesses of the PEI and PI matrices on the TF formation and adherence on the sliding surfaces of the composites with both crystalline and polymeric solid lubricant microparticles were assessed at room and elevated temperatures.

1. In the tribological tests of the ternary PEI-based composites at room temperature, the counterpart materials (steel or ceramic) did not significantly affect the tribological properties, namely their both CoF and WR.
2. Loading the PEI- and PI-based composites with PTFE caused sliding in the ‘wearless’ mode due to the easy separation of PTFE flakes with the subsequent formation of the thin continuous transfer films on the composite friction surfaces at the CoF levels of 0.05–0.11 (at room temperature). With an increase in the test temperature, the average CoF level and the amplitude of its oscillations increased for both PEI- and PI-based composites. This prevented the transfer film from adhering to the wear track surfaces of the PTFE-containing PI-based samples at *T* = 180 °C. For the similar PEI-based ones, their more pliable matrix enabled to provide the extremely low wear at the levels of less than 0.2 × 10^−6^ mm^3^/N m with the increased CoF values of 0.11.
3. Loading of MoS_2_ into the reinforced “PI-CCF” and “PEI-CCF” composites ensured the ‘solid lubricant’ sliding mode at the low CoF level of 0.1, which demanded for stimulating oxidation process (tougher friction conditions, primarily due to the increase in the test temperature and enlarging the CoF). However, it was not possible to reduce the WR values below 2 × 10^−6^ mm^3^/N m due to the difference in the solid lubrication mechanisms ensured by crystalline MoS_2_ compared to polymer PTFE.
4. Raising the MoS_2_ content up to 23 wt.%, which was identical in volume to other used solid lubricant fillers (PTFE and Gr), made it possible to decrease the CoF levels for the PEI- (at *T* = 180 °C) and PI-based (at *T* = 120–240 °C) composites via the formation and adhering the transfer films. However, this effect was not realized at room temperature because of the low intensity of oxidative processes.
5. Under the applied tribological conditions, colloidal graphite could not be considered as a solid lubricant filler for the studied ternary PEI- and PI-based composites, since the high interlayer shear energy did not ensure the formation of an anti-friction film on the wear track surfaces, maintaining the high both CoF and WR levels with rising the test temperature.
6. The comparative analysis of the behavior of the PEI- and PI-based composites at the elevated temperatures showed that the WR values for the PEI-based samples were lower by 300 times than those for the PI-based ones at *T* = 180 °C.
7. The PEI-based composites are recommended for use in tribological units both due to their high manufacturability because of the flexibility of polymer chains, and great wear resistance via the formation of fixed continuous transfer films on the wear track surfaces in the entire investigated temperature range of 23–180 °C. The PI-based composite containing 23 wt.% MoS_2_ (PI/10CF/23MoS_2_) might efficiently operate in tribological units at the elevated temperatures 180 ÷ 240 °C.

## Figures and Tables

**Table 1:** The crystallinity degrees of the initial PEI and PI powders.

Sample	Crystallinity, %
PEI ROOH	32
PI 1600	21

**Table 2:** The compositions and the glass transition temperatures of the investigated composites.

Filler Content, vol.%	T_g_, °C	Filler Content, wt.%	Designation
PI	259	PI	PI
PI + 8.3%CF	265	PI + 10%CF	PI/10CCF
PI + 8.3%CF + 6.6%PTFE	266	PI + 10%CF + 10%PTFE	PI/10CCF/10PTFE
PI + 8.3%CF + 6.6%Gr	259	PI + 10%CF + 10%Gr	PI/10CCF/10Gr
PI + 8.3%CF + 3.0%MoS_2_	256	PI + 10%CF + 10%MoS_2_	PI/10CCF/10MoS_2_
PI + 8.3%CF + 6.6%MoS_2_	256	PI + 10%CF + 23%MoS_2_	PI/10CCF/23MoS_2_
PEI	211	PEI	PEI
PEI + 8.3%CF	212	PEI + 10%CF	PEI/10CCF
PEI + 8.3%CF + 6.6%PTFE	211	PEI + 10%CF + 10%PTFE	PEI/10CCF/10PTFE
PEI + 8.3%CF + 6.6%Gr	215	PEI + 10%CF + 10%Gr	PEI/10CCF/10Gr
PEI + 8.3%CF + 3.0%MoS_2_	215	PEI + 10%CF + 10%MoS_2_	PEI/10CCF/10MoS_2_
PEI + 8.3%CF + 6.6%MoS_2_	215	PEI + 10%CF + 23%MoS_2_	PEI/10CCF/23MoS_2_

**Table 3:** The physical and mechanical properties of neat PEI and the PEI-based composites.

No.	Filler Composition (wt.%)	Density *ρ*, (g/cm^3^)	Shore *D* Hardness	Elastic Modulus E (GPa)	Ultimate Tensile Strength σ_U_ (MPa)	Elongation at Break ε (%)	Stored Energy (kJ/m^3^)
1	Neat PEI	1.26	79.9 ± 0.3	3.12 ± 0.15	123.1 ± 0.5	16.1 ± 1.2	14,700
2	PEI/10CCF	1.31	81.4 ± 0.3	6.54 ± 0.43	153.2 ± 12.5	3.7 ± 0.6	2900
3	PEI/10CCF/10PTFE	1.36	79.0 ± 0.3	6.17 ± 0.26	117.3 ± 8.0	3.1 ± 0.3	1900
4	PEI/10CCF/10Gr	1.36	80.6 ± 0.2	6.37 ± 0.16	101.4 ± 2.6	2.8 ± 0.1	1500
5	PEI/10CCF/10MoS_2_	1.41	81.9 ± 0.1	6.26 ± 0.17	121.0 ± 5.0	3.5 ± 0.3	2200
6	PEI/10CCF/23MoS_2_	1.56	81.9 ± 0.3	6.81 ± 0.14	101.4 ± 1.7	2.5 ± 0.1	1400

**Table 4:** The tribological characteristics of the PEI-based composites (*P* = 5 N; *V* = 0.3 m/s).

Composite	Coefficient of Friction ƒ		Wear Rate (10^−6^ mm^3^/N · m)	
	Metal Counterpart	Ceramic Counterpart	Metal Counterpart	Ceramic Counterpart
Neat PEI	0.297 ± 0.026	0.342 ± 0.032	390.21 ± 20.51	443.72 ± 24.90
PEI/10CCF	0.286 ± 0.018	0.270 ± 0.009	30.39 ± 2.68	15.20 ± 1.23
PEI/10CCF/10PTFE	0.045 ± 0.005	0.048 ± 0.004	0.45 ± 0.04	0.31 ± 0.03
PEI/10CCF/10Gr	0.228 ± 0.016	0.245 ± 0.005	14.7 ± 1.35	4.83 ± 0.43
PEI/10CCF/10MoS_2_	0.238 ± 0.014	0.253 ± 0.010	5.63 ± 0.52	5.04 ± 0.48
PEI/10CCF/23MoS_2_	0.248 ± 0.012	0.261 ± 0.023	8.13 ± 0.87	7.52 ± 0.62

**Table 5:** The results of EDS analysis of the transfer film and debris on the PEI-composites in accordance with the labels in Figure 6.

Element	Spectrum 1 wt.%/at.%	Spectrum 2 wt.%/at.%	Spectrum 3 wt.%/at.%	Spectrum 4 wt.%/at.%	Spectrum 5 wt.%/at.%
PEI/10CCF/10PTFE					
C	73.77/78.32	81.65/85.98	51.61/63.56	48.49/59.59	80.06/84.77
O	26.23/21.68	18.35/14.02	7.92/4.92	5.21/4.43	19.94/15.23
F			40.47/31.52	46.30/35.98	46.1/35.5
PEI/10CCF/10Gr					
C	81.16/85.16	81.58/85.51	85.11/89.47	79.17/83.51	78.79/83.19
O	18.84/14.84	18.42/14.49	14.89/10.53	20.83/16.49	21.21/16.81
PEI/10CCF/10MoS_2_					
C	69.52/83.80	72.56/84.61	61.92/82.95	81.25/86.70	76.85/88.52
O	13.06/11.82	13.78/12.06	8.74/8.79	15.85/12.70	9.55/8.26
Mo	11.57/1.75	9.08/1.33	19.35/3.25	2.08/0.28	9.23/1.33
S	5.85/2.64	4.58/2.00	9.99/5.01	0.82/0.33	4.37/1.88

**Table 6:** The tribological properties of the PEI-based composites (*P* = 5 N; *V* = 0.3 m/s).

T, °C	PEI/10CCF/10PTFE		PEI/10CCF/10Gr		PEI/10CCF/10MoS_2_		PEI/10CCF/23MoS_2_	
	Coefficient of Friction	Wear Rate (10^−6^ mm^3^/N·m)	Coefficient of Friction	Wear Rate (10^−6^ mm^3^/N·m)	Coefficient of Friction	Wear Rate (10^−6^ mm^3^/N·m)	Coefficient of Friction	Wear Rate (10^−6^ mm^3^/N·m)
23	0.048 ± 0.004	0.31 ± 0.03	0.245 ± 0.005	4.83 ± 0.43	0.253 ± 0.010	5.04 ± 0.48	0.261 ± 0.023	7.52 ± 0.62
120	0.088 ± 0.009	0.20 ± 0.02	0.439 ± 0.015	126.35 ± 9.85	0.403 ± 0.030	39.07 ± 3.70	0.234 ± 0.017	9.13 ± 0.76
180	0.107 ± 0.012	0.24 ± 0.02	0.452 ± 0.031	163.98 ± 13.16	0.095 ± 0.011	9.36 ± 0.95	0.111 ± 0.009	2.15 ± 0.23

**Table 7:** The results of EDS analysis of the transfer films and debris on the PEI-based composites in accordance with the labels in Figure 8. A test temperature of 180 °C.

Element	Spectrum 1 at.%	Spectrum 2 at.%	Spectrum 3 at.%	Spectrum 4 at.%	Spectrum 5 at.%
PEI/10CCF/10PTFE					
C	43.93	61.55	62.23	61.72	65.49
O	12.38	22.17	27.55	18.31	21.45
F	43.69	16.28	10.22	19.97	13.06
PEI/10CCF/10Gr					
C	75.57	64.32	73.83	72.65	70.37
O	24.43	35.68	26.17	27.35	29.63
PEI/10CCF/10MoS_2_					
C	77.24	69.45	70.40	68.68	82.17
O	8.90	26.54	26.39	9.95	14.69
S	9.67	2.65	2.16	15.08	2.10
Mo	4.19	1.36	1.04	6.30	1.05
PEI/10CCF/23MoS_2_					
C	68.58	72.56	61.92	81.25	76.85
O	10.73	13.78	8.74	15.85	9.55
S	13.84	9.08	19.35	2.08	9.23
Mo	6.85	4.58	9.99	0.82	4.37

**Table 8:** The tribological properties of the PI-based composites (*P* = 5 N; *V* = 0.3 m/s).

T, °C	PI/10CCF/10PTFE		PI/10CCF/10Gr		PI/10CCF/10MoS_2_		PI/10CCF/23MoS_2_	
	Coefficient of Friction	Wear Rate (10^−6^ mm^3^/N·m)	Coefficient of Friction	Wear Rate (10^−6^ mm^3^/N·m)	Coefficient of Friction	Wear Rate (10^−6^ mm^3^/N·m)	Coefficient of Friction	Wear Rate (10^−6^ mm^3^/N·m)
23	0.094 ± 0.007	0.27 ± 0.02	0.226 ± 0.023	2.60 ± 0.23	0.295 ± 0.021	23.71 ± 2.31	0.262 ± 0.022	43.66 ± 1.90
120	0.182 ± 0.019	0.25 ± 0.03	0.394 ± 0.040	141.79 ± 15.14	0.498 ± 0.045	65.96 ± 6.81	0.082 ± 0.011	2.82 ± 0.29
180	0.320 ± 0.050	72.49 ± 7.12	0.397 ± 0.099	372.30 ± 36.82	0.519 ± 0.046	140.27 ± 13.56	0.076 ± 0.013	3.93 ± 0.38
240	0.270 ± 0.077	68.35 ± 6.43	0.395 ± 0.165	542.88 ± 68.34	0.513 ± 0.060	284.92 ± 29.31	0.161 ± 0.069	1.95 ± 0.17

**Table 9:** The results of EDS analysis of the transfer films and debris on the PI-based composites in accordance with the labels in Figure 10. The test temperature of 180 °C.

Element	Spectrum 1 at.%	Spectrum 2 at.%	Spectrum 3 at.%	Spectrum 4 at.%	Spectrum 5 at.%
PI/10CCF/10PTFE					
C	55.60	89.76	92.69	57.93	58.12
O	31.84	7.44	5.16	23.46	38.06
F	12.56	2.80	2.15	18.61	3.82
PI/10CCF/10Gr					
C	65.88	92.99	80.74	64.67	88.96
O	34.12	7.01	19.26	35.33	11.04
PI/10CCF/10MoS_2_					
C	67.82	69.40	74.38	82.97	73.83
O	24.23	24.17	17.36	11.48	15.12
S	6.38	5.32	6.37	4.70	9.23
Mo	1.57	1.11	1.89	0.85	1.82
PI/10CCF/23MoS_2_					
C	66.82	-	-	62.96	94.02
O	22.07	61.71	50.98	21.13	5.27
S	11.11	38.29	49.02	12.57	0.71
Mo	-	-	-	3.34	-

**Table 10:** Friction coefficients and specific wear rates of PEI- and PI-based Composites.

Material	Contact Type and Counterpart Material	Working Conditions (*T*_Rt_–Room Temperature)		Friction Coefficient	Specific Wear Rate [10^−6^ mm^3^/N · m]	Ref.
5 vol.% micro-CaSiO_3_/10 vol.% Gr/15 vol.% SCF/PEI	Pin-On-Disc, Metal counterpart (Ra = 0.1 μm)	*P* = 1 MPa *V* = 1 m/s	*T*_Rt_ *T* = 150 °C	~0.18–0.22 ~0.12	0.20–0.29 1.67–1.74	[32]
5 vol.% nano-Gr/10 vol.% Gr/15 vol.% SCF/PEI			*T*_Rt_ *T* = 150 °C	~0.2 ~0.05	0.29 0.95	
5 vol.% Gr/15 vol.% SCF/PEI	Pin-On-Disc, Metal counterpart (Ra = 0.22 μm)	*P* ∈ [1,12] MPa *V* ∈ [1,3] m/s	*T*_Rt_ *T* ∈ (70,120) °C	0.22–0.61 0.24–0.29	0.73–598.67 1.92–15.18	[55]
5 vol.% nano-TiO_2_/5 vol.% Gr/15 vol.% SCF/PEI			*T*_Rt_ *T* ∈ (70,120) °C	0.09–0.36 0.09–0.26	0.30–2.99 1.08–29.27	
5–20 vol.% SCF/PEI	Pin-On-Disc, Metal counterpart	*P* = 2 MPa *V* = 1 m/s	*T*_Rt_ *T* = 150 °C	~0.35 ~0.15–0.25	~0.8 ~5	[13]
20 vol.% PBO_RES-treated_ /PI	Ball-On-Disc, Metal counterpart (Ra = 0.1 μm)	*F* = 6 N *V* = 0.5 m/s	*T*_Rt_ *T* = 210 °C	0.35 0.2	5 22	[66]
1.5 wt.% SMPS/PI	Ball-On-Disc, Metal counterpart	*F* ∈ [5,15] N *V* = 0.08 m/s	*T*_Rt_ *T* ∈ (100,300) °C	~0.13–0.40 ~0.15–0.42	~0.75–1.60 ~0.7–2.4	[54]
5–30 vol.% CF/PI	Ball-On-Disc, Metal counterpart (Ra = 0.02 μm)	*F* = 5 N *V* = 0.3 m/s	*T*_Rt_ *T* ∈ (100,260) °C	~0.30–0.32 ~0.05–0.37	~5.7–9.1 ~3.4–29.5	[57]
15 vol.% CF/PI	Block-On-Ring, Metal counterpart (Ra = 0.1 μm)	*F* = 30 N *V* = 1 m/s	*T*_Rt_ *T* ∈ (50,200) °C	0.30 0.32–0.35	~2.2 ~7.5–21	[58]
15 vol.% GF/PI			*T*_Rt_ *T* ∈ (50,200) °C	0.43 0.47–0.53	~2.0 ~4–13	
15 vol.% AF/PI			*T*_Rt_ *T* ∈ (50,200) °C	0.34 0.30–0.38	~10 ~11–55	
20 vol.% PTFE /PI	Cylinder-On-Plat, Metal counterpart (Ra = 0.05 μm)	*F* ∈ [50,200] N *V* ∈ [0.3, 1.2] m/s	*T*_Rt_ *T* ∈ (50,260) °C	~0.12–0.30 ~0.10–0.22	~65–78 ~70–120	[27]
30 wt.% CF/PI	Cylinder-On-Plat, Metal counterpart (Ra = 0.05 μm)	*F* = 50 N *V* = 0.3 m/s	*T*_Rt_ *T* ∈ (60,260) °C	~0.55 ~0.05–0.65	~40 ~80–600	[60]
15 wt.% PTFE/30 wt.% CF/PI			*T*_Rt_ *T* ∈ (60,260) °C	~0.2 ~0.05–0.23	~15 ~102–700	
15 wt.% silicon oil /30 wt.% CF/PI			*T*_Rt_ *T* ∈ (60,260) °C	~0.7 ~0.24–0.92	~20 ~105–800	
**10 wt.% PTFE/10 wt.% CF/PEI**	**Ball-On-Disc,** **Ceramic counterpart** **(Ra = 0.02 μm)**	***F*** **= 5 N** ***V* = 0.3 m/s**	***T*** **_Rt_** ***T*** **∈** **(120,180) °C**	**0.05** **0.09–0.11**	**0.31** **0.20–0.24**	**Present work**
**23 wt.% MoS_2_/10 wt.% CF/PI**			***T*** **_Rt_** ***T*** **∈** **(120,240) °C**	**0.262** **0.08–0.16**	**44.0** **2.0–4.0**	

## Data Availability

The data presented in this study are available on request from the corresponding author.
